# Surgical Treatment

**Published:** 1951-10

**Authors:** R. Milnes Walker


### II—SURGICAL TREATMENT

BY

R. MILNES WALKER

---

The surgical treatment of disorders of the heart has been one of the startling and sensational events in medical practice of the century. Yet in actual fact the story only goes back twelve years: for it was in 1939 that Gross in Boston successfully ligatured a patent ductus arteriosus, an operation which proved to be the advent of great things to come. It is not my intention here to go into the surgical details of the operations which are nowadays performed for congenital abnormalities of the heart and great vessels, but to say a word about the indication for operation and to give you some idea of the immediate results which are to be expected. As no patient has yet had the opportunity of surviving an operation for patent ductus arteriosus more than twelve years, or for coarctation of the aorta or cyanotic heart disease more than six years, any statement about long-term results would be only a surmise. I think it is safe to say there is every indication that, in the case of patent ductus or coarctation of the aorta, the patient who survives a successful operation has the prospect of a normal span of life, provided complications had not already set in before the operation was undertaken. And this brings me to a final word in general and that is that there is an optimum time in life for dealing with these conditions and this may be before the patient has any symptoms: for the complications creep in insidiously and many of them may soon reach a state which is irreversible and may thus render the operation more hazardous and the ultimate prognosis more serious.

*Patent ductus arteriosus.* There are two operations employed for this condition, either ligature of the ductus or else its actual division with suture of the two openings remaining in the aorta and pulmonary artery. In this country the application of double ligatures, one at each end of the ductus, is usually employed as being the safer operation and giving satisfactory results with only a very slight risk of recanalisation. Many American centres adopt the more radical procedure of division, but this requires higher technical skill and it is doubtful if the results justify the additional dissection which is necessary. Crafoord in Stockholm adopts an intermediate course, ligaturing most of them but dividing the short wide ones.

The best age for operation is about five or six years, and any evidence of commencing cardiac dilatation renders operation an urgent matter. It is my own view that in the absence of other cardiac abnormalities all cases should be operated on about this age whether there are symptoms or not, for it has been shown (Keys and Shapiro, 1943) that people who are alive at the age of seventeen with a patent ductus have a life expectancy which is only half that of the general population. The operation, if carried out in the absence of complications, is safe and in itself causes no disability. It is one of the most satisfying operations in surgery for it is one of the few which convert an abnormal condition into one which is completely normal and physiological.

My own series comprises twenty-four cases with no mortality; in only one case was there a serious complication, for I accidentally tore a hole in the pulmonary artery and to close this necessitated division of the ductus; unfortunately I included the recurrent laryngeal nerve in a clamp and the resultant paralysis of the vocal cord made it necessary to perform a tracheotomy which had to be retained for about a week, but the child eventually made a complete recovery.

There is an interesting point about another patient; she is the oldest patient in the group, twenty-two years, but the point is that her mother had died as a result of a patent ductus which became infected during the puerperium after this patient was born, and we managed to obtain a full account of the autopsy performed at the London Chest Hospital over twenty years ago.

*Coarctation of the aorta.* As with a patent ductus, many patients with this abnormality live to adult life without great disability but the expectation of life is much reduced and complications may occur without warning. From the surgeon's point of view the main anxiety is that atheroma develops in the wall of the aorta at an unusually early age and thus renders it friable and sutures in it will not hold. This is therefore an argument for early operation; one of our patients, aged seven, already had early atheromatous changes in the wall of the arch of the aorta, and most surgeons are agreed that the hazards of the operation make it unjustifiable after the age of thirty.

The present operation was devised by Crafoord in Stockholm, and first reported in 1945. His operation consists of excision of the stricture and performing an end-to-end anastomosis of the aorta. We have modified this slightly in that I do not excise the whole circumference of the stricture but take out a crescent of about two-thirds of the circumference and leave a bridge of aorta on the inner side so that the whole strain of maintaining the ends in apposition does not fall on the sutures.

Reported series of cases are as follows:

| | Cases explored | Coarctation excised | Deaths | Mortality per cent | Later Complications |
|---|---|---|---|---|---|
| Crafoord (1951) | 85 | 75 | 6 | 8 | 4 aneurysms |
| Gross (1950) | 100 | 91 | 11 | 12 | 1 fatality from aneurysm |

In six of his patients Gross inserted a graft of human aorta to fill a gap left after the excision and these patients have survived from three months to one-and-a-half years satisfactorily.

Our own cases are listed below:

| | Age | Sex | Systolic B.P. | Complications before operation | Operation result |
|---|---|---|---|---|---|
| 1. M.E. | 12 | M | 160 | | Well 3½ years |
| 2. I.D. | 12 | M | 160 | Intracranial haemorrhage | Well 3 years B.P. 110 |
| 3. B.C. | 7 | M | 140 | | Died—acute tracheo-bronchitis |
| 4. P.S. | 8 | M | 180 | | Well 6 months B.P. 120 |
| 5. M.P. | 23 | F | 220 | | Well 4 months B.P. 140 |
| 6. S.P. | 11 | F | 150 | Rheumatic mitral stenosis | Died—rupture of aorta 5th day |
| 7. W.L. | 10 | M | 190 | | Satisfactory progress 2 weeks B.P. 155 |

It is my view that operation should be carried out on all patients under twenty who have symptoms or who have a rising blood pressure, unless there is some complication which will make the operation particularly dangerous; it should preferably be undertaken before the age of ten.

*Cyanotic heart disease.* The credit for instituting surgical means by which additional blood may be brought to the lungs for oxygenation in congenital heart disease goes to Helen Tausig and Alfred Blalock of Johns Hopkins Hospital in Baltimore, and as with coarctation their first results were published in 1945. They felt that if some of the blood in the systemic circulation could be shunted back into the pulmonary circuit this result would be achieved: and the method adopted was to anastomose one of the subclavian arteries to the corresponding pulmonary artery. Potts of Chicago attained the same result by a direct side to side anastomosis between the descending part of the arch of the aorta and one of the pulmonary arteries, this forming in effect a patent ductus arteriosus. These operations do not, of course, overcome any of the abnormalities existing in the heart and in fact add one more, but the results fully justified the procedure. It is still too early to assess the long-term results, but in Fallot's Tetralogy the change in the colour and activity of the patient is in the majority of cases dramatic.

Cases of pure pulmonary stenosis do not respond so well to a Blalock or Potts operation and for them Brock has devised a direct attack on the site of the obstruction, approaching it through the right ventricle and, in the valvular type, cutting the diaphragm with a knife to make a bicuspid valve, or in the infundibular type punching out part of the obstruction. The results have been most encouraging, and here the actual lesion is being attacked, obviously an improvement on the bypass operation which in fact adds a second abnormality. Brock has carried out in America valvulotomies on some patients who had previously been submitted to a Blalock operation without benefit and after the valvulotomy the Blalock anastomosis has been closed. He is also applying valvulotomy in cases of Fallot's Tetralogy with encouraging results, but how they will compare in the long run remains to be seen. These considerations will convince you how important is an accurate diagnosis before operation.

The following are samples of results which have been reported:

| | | *Operation* | *Cases* | *Deaths* | |
|---|---|---|---|---|---|
| Brock | Pulmonary stenosis | Valvulotomy | 28 | 8 | 4 before heart was touched |
| | Fallot's Tetralogy (only 1 death in last 29) | Valvulotomy | 29 | 2 | |
| | Fallot's Tetralogy | Infundibular resection | 24 | 7 | |
| Sellors (1950) | Cyanotic heart disease | Blalock operation | 93 | 9 | |

My own experience of cases of cyanotic heart disease is limited and insufficient to provide any conclusions; the first cases were extremely ill and we had some disappointing results. It is now clear that the surgeon must have at his command the experience and the instruments to direct his attack to the lesion in the pulmonary artery or infundibulum or to make a vascular anastomosis outside the heart. Each case must be judged individually and the correct procedure may not be determined until the pericardium is open and the heart can be inspected and palpated. The view is generally accepted that the risk of any of these operations greatly increases as the years pass, and as with the other abnormalities which I have discussed, operation should be undertaken during the second half of the first decade of life.

*Experimental work on septal defects.* There has been a good deal of enquiry into surgical methods of closing defects in the cardiac septa, but as they have not yet reached the stage of regular clinical practice I will not say anything further about them now.

The number of cases of cyanotic heart disease which will benefit by surgical treatment is not very great and they should be concentrated in a limited number of centres. Recent developments of intracardiac surgery in the treatment of acquired mitral stenosis suggest that this is in fact a matter of much greater magnitude as regards the number of patients to be treated, and that during the next five years it will materially improve their prognosis quite as dramatically as the outlook has changed for those with congenital cardiac defects during the past decade.

REFERENCES

Brock, R. C. (1951)—*B.M.Jl.*, i, 946.

Crafoord, C. (1951).—*B.M.Jl.*, 1951, i, 946.

Gross, R. E. (1950).—*Circulation*, **1**, 41.

Keys and Shapiro (1943).—*Amer. Heart Jl.*, **25**, 158.

Sellors, T. H. and Belcher, J. R. (1950).—*Lancet*, 1950, ii, 887.

